# How to Promote Workplace Health in order to Work into Old Age: Experiences from Employees in an Industrial Setting

**DOI:** 10.1155/2019/3942569

**Published:** 2019-04-01

**Authors:** Marie Lydell, Cathrine Hildingh, Arne Söderbom, Kristina Ziegert

**Affiliations:** ^1^School of Social and Health Sciences (Akademin för hälsa och välfärd), Halmstad University, Halmstad, Sweden; ^2^Research on Welfare, Health and Sport (Centrum för välfärd, hälsa och idrott), Halmstad University, Halmstad, Sweden; ^3^School of Business, Engineering and Science (Akademin för ekonomi, teknik och naturvetenskap), Halmstad University, Halmstad, Sweden; ^4^Center for Innovation, Entrepreneurship and Learning Research (Centrum för innovations-entreprenörskaps-och lärandeforskning), Halmstad University, Halmstad, Sweden

## Abstract

**Background:**

Health is important in workplaces. A good organisational climate in a workplace plays a major role in the employees' well-being at work and is also associated with increased productivity. Today, employees are expected to work into older age and it is a challenge for companies to promote health and well-being for this growing group.

**Purpose:**

The purpose of this study was to explore how to promote workplace health at present time and for the end of working life in the perspective of employees.

**Design/Methodology/Approach:**

The study had an explorative design, and a thematic analysis was chosen. The inclusion criteria were persons 50 years and older, working in the company and planning to continue working into old age. A total of 21 coworkers (14 men) participated in the study. Three focus group interviews were conducted.

**Findings:**

The results from the focus group interviews are presented in four themes: handle change in a changeable workplace, take responsibility for health concerns, get confirmation for feeling needed, and support and tolerance adapted for each employee.

**Practical Implications:**

Promoting health should be an urgent mission for employees themselves as well as for managers in order to support employees in working into old age. The health promotion must be suitable for each employee and should be designed in such a way as to avoid inequality in workplace health.

**Originality/Value:**

There is a need for more health-promoting behaviours, support, and activities for employees in order to work into old age.

## 1. Introduction

All western countries are seeking to bring retirement ages more in line with increases in longevity. People have expectations about their remaining length of life, and these expectations have strong impacts on social, economic, and health resources [1]. Although health is a complex concept to define, it needs to be done. There is a pathogenic perspective and a salutogenic perspective to health, and the combination of these perspectives is of great importance when dealing with workplace health. With a pathogenic perspective, the focus is directed on curing and preventing illness, and this is the most common perspective within the healthcare sector. With a salutogenic perspective, the focus is not on preventing disease, but on maintaining or increasing health [2]. Sense of coherence (SOC) is a part of the salutogenic theory and consists of the concepts of comprehensibility, manageability, and meaningfulness. SOC theory is used as a guide for health promotion because it highlights opportunities and identifies resources instead of focusing on risk factors for diseases [3]. Preventing illness, as well as promoting health, is the mission for the occupational health service (OHS). However, both the staff at occupational health service centres (OHSCs) and managers in companies have expressed their need for more health-promoting interventions instead of just preventing illness in workplaces [4, 5].

Workplace health promotion has been defined as “the combined efforts of employers, employees, and society to improve the health and well-being of people at work” [6]. Feldt et al. [7] suggested that a good organisational climate in a workplace plays a major role in supporting SOC, which influences the employee's well-being at work.

Moreover, good health is important in workplaces because increased employee health at work is associated with increased productivity [8]. Regarding older employees, SOC is correlated with physical, social, and mental well-being [9]. Furthermore, it has been shown that working into older age is good for one's health, although the type of job seems to be a critical factor in this, and undesirable jobs can negate the potential favourable effects of work [10]. Even though work at older ages seems beneficial for many, the benefits might decrease or stop increasing, after a certain age or amount of time worked per year [10].

There are many challenges that must be dealt with at the workplace, and one of them is the rapid increase in the elderly population and the expectation that employees will work longer [11, 12]. Another challenge is that there could be tensions between older and younger employees, for example, regarding technology issues in the workplace [13, 14]. This issue might cause stress in older employees, and this can lead to problems between younger and older employees and can affect workplace health. When employees are expected to work into older ages, it is important to find effective ways to promote health for this particular group. It is also important to assess the experiences of employees getting older within a workplace in order to ensure equal health opportunities for these groups. Therefore, the aim of the study was to explore how to promote workplace health at present time and for the end of working life in the perspective of employees.

## 2. Materials and Methods

### 2.1. Design

A qualitative exploratory design with thematic analysis was chosen in order to explore employees' experiences of how to promote workplace health at present time and for the end of working life [15].

### 2.2. Participants

The study took place in an industrial setting in the western part of Sweden through purposive selection. This means that members of a sample population are chosen with a purpose to represent specific persons in relation to the criterion [16]. The sample population was people working in the company, and the specific persons should be 50 years and older, working in the company, and planning to continue to work into old age. All employees in the company over 50 years old (*n*=21) received oral and written information regarding the study and were invited by the manager of the company to participate in focus group interviews. A total of 21 employees agreed to participate in the study, including 14 men and 7 women. The participants were between 50 and 64 years old and consisted of both white collar and blue collar workers.

### 2.3. Data Collection

Three focus group interviews, with seven persons in each group, were conducted at their current workplace. Focus groups were chosen in order to take advantage of group dynamics in order to access rich information [17]. Specifically, the group's social dynamic was expected to generate a chain of conversational arguments based on distinct knowledge on the need for health support in a workplace setting. An interview guide was developed, and it included questions about their experiences of how workplace health can be promoted. Examples of the questions included: How can health at the workplace be promoted for older employees? What does the company do to promote health for older employees? What do you do to promote health? What needs have you experienced for promoting health in the workplace? Probing questions were also asked, such as, Can you give an example of actions for health promotion at work? A moderator led the discussions together with a comoderator who stimulated the participants to take part in the interview and took notes about any nonverbal communication during the discussion. The focus group interviews were audio-recorded and lasted 50–60 minutes.

### 2.4. Data Analysis

The data were analysed by using thematic analysis according to Braun and Clarke [15]. An inductive approach was chosen for the focus group interviews in order to capture the coworkers' thoughts and experiences of promoting workplace health. The analysis started with reading the material several times to get familiar with the data set and to search for patterns. In the next step, initial codes were identified in the data that were relevant for the aim of the study. While re-reading the codes, five themes emerged, and each text unit was retested to ensure that it was accurately classified in the respective theme. The five themes were then reviewed by cross-checking the entire data set, and this resulted in the final four themes presented here. The analysis was performed and discussed throughout the whole process by all of the authors (Table 1). Quotations from different informants have also been chosen to illustrate the findings.

### 2.5. Ethical Consideration

This study was supported by the Knowledge Foundation and has undergone a review process including ethical review (KKS dnr 20130309). The ethical issues were reflected upon to ensure that any potential harm was minimised. The autonomy and integrity of the participants were respected, and the principles of the Declaration of Helsinki were followed, including the confidential procedures for requesting participation and for storing the collected material. Written information about the project was sent to the participants in the study, including information that participation in the study was voluntary and could be discontinued at any time. Written and verbal consent was obtained by all of the participants. Confidentiality was guaranteed, and all material was treated according to the Swedish law on personal data (Personuppgiftslagen, 1998 : 204). The data material was coded with nonidentifiable codes, was stored in locked cabinets, and only the researchers had access to the data.

## 3. Results and Discussion

### 3.1. Results

Results from the focus group interviews are presented in four themes regarding employees' experiences of how to promote health in workplaces—*handle change in a changeable workplace*, *take responsibility for health concerns*, *get confirmation for feeling needed,* and *support and tolerance adapted for each employee*.

#### 3.1.1. Handle Change in a Changeable Workplace

There had been many changes in the workplace in recent years, and the coworkers were of the opinion that this was both good and bad. In some periods, the employees had got several new duties every week and they never knew exactly what to do. If this was to continue, it could be hard for the coworkers in the future when they were getting older. There had also been a reorganisation in the company, which resulted in that many of the employees were quitting. One way to feel good at work was if they had access to temporary staff. If not, the employees had to handle the same job with fewer persons, and therefore, they would have no time for breaks and reflection. This is an issue to handle in the future and could be hard when getting older. The stress that arises must be taken care of, and the employees reported having different strategies for doing so. The workplace has its own gym for employees, and some of them were training there to overcome the stress. One idea discussed was that there is a need for coaches who can help the employees to handle changes in order to prevent illness and promote health. However, strategies for handling the changes had been discussed at a workplace meeting, and the employees were aware that adaptation for these changes had to be made for both older and younger employees.

To promote health in the workplace, the employees felt that they would have to change duties when getting older. They said that it is hard to learn about new technology and that the brain may not be so quick any more in old age.

“You have to keep up with both the technology and the thoughts are not as quick as before. When the brain works slower, it is harder to understand new things.”

However, new technology can often actually help older employees if they understand how to use it. This must be prepared for, and learning in a calm environment was suggested to be necessary for successful learning. Another problem that came up in the group discussions was that there is a fast pace and sometimes a hard workload that might be difficult to handle when getting older. For promoting health, this issue should be taken care of by the managers. Before anything happens, the employees also expressed that there must be more safety inspections and ergonomic inspections. There was a need for discussions regarding work-life balance, and the participants wanted support from safety inspections and ergonomic inspections and support from the OHSC.

Another factor emphasised by the employees was that the company's customers had become more stressful and were often in a hurry when demanding services and support. E-mail was also an important stressor, and the employees said that they would need less e-mail conversations when they get older because then “you are more affected by stress.” Some of the employees had decided that they would work fewer hours when they get older, but they were concerned about the risk that they might miss what happened while they were not at work and how this could also be stressful.

“If you work less time you miss what happens, and there is a risk for being outside the group.”

#### 3.1.2. Take Responsibility for Health Concerns

The employees were of the opinion that everyone has the responsibility for their own health.

“Every employee is responsible for taking care of their health and this could be done in different ways. I like to take some walks after work.”

To prevent illness and promote health at work, they need to move and engage in exercise. The younger employees were training more often and harder than the older ones, and some of the participants suggested that the employees might need guidance regarding good exercise routines. They also expressed that it would be easier to train if they got some kind of wellness grant. To take breaks and stand up instead of sitting the whole day was another suggested way to promote health.

They were aware that health is much more than training, and they said that it is very important to find a balance between work and leisure time. The opinion from the employees was that it is both a personal responsibility as well as the manager's responsibility to help them find such a balance.

Many of the employees were accustomed to going to work even if they were sick. They also expressed that working while sick might be a generational question, and they thought this was different for the younger employees.

“We have always worked even if we were sick, but the younger employees haven't.”

They thought that working while sick is about relations to one's coworkers and staying home sick would leave more work for their coworkers. They said that taking responsibility for the work to be done was of greater importance than taking responsibility for their own health. The idea of putting their coworkers in a stressful situation for having to do their job for them made the choice to be at work while sick an easy choice.

#### 3.1.3. Get Confirmation for Feeling Needed

To have good health at work, the individuals must feel that they are important in the working team and are seen and confirmed as a part of the team. The employees meant that every employee must be confirmed by other employees and by the managers. Everyone has a need for confirmation, the elderly as well as the younger. They also said that feeling needed at work creates a personal value that promotes the individual's health. Having a good work climate promoted health, according to the coworkers, and therefore, managers have the responsibility to help them to understand that they are doing a good job.

“The managers should let us know that we are doing a good job, it is important that we are being told.”

Helping customers makes them feel good and even more so if the customers are thankful. This is a prerequisite for enjoying working, being confirmed, and feeling comfortable at the workplace. If one does not enjoy being at work, then one will not work that long in the workplace and will instead retire from work.

“Not enjoying work, or working, and if you have the possibility to quit, you do.”

The employees were of the opinion that it is, of course, the manager's responsibility to let the employees know that they are important, but also that employees let each other know that they are important. Furthermore, they discussed that to hug someone is very important for feeling good during the workday and that it is necessary for knowing that we see each other and are needed in the daily work.

“If every person got seven hugs a day, everyone would feel good, but how many get that?”

The employees had participated in a course about different personalities and how to act in different situations in order to understand each other better. This could be a developmental process, both for the team and individually, which was experienced as promoting their own health.

There were some experiences from the employees regarding sick leave due to stress. The OHSC helped the employees to manage going back to work, and there was also a plan for handling stressful situations. This had strengthened and promoted their health, and today the employees felt comfortable at work. This would also help the employees to feel secure about the future, knowing that if they will be on sick leave there is support and the ability to get help.

#### 3.1.4. Support and Tolerance Adapted for Each Employee

To get older requires both support and tolerance from the management, and the employees had both positive and negative experiences regarding this. Some of them said that they had support from both the management and the OHSC, which made them feel good. However, they sometimes missed the sense of safety and experienced a lack of information about what was happening in the company, and this could be a hindrance for health promotion in the workplace. When this happened, thoughts about retiring from work came up, but the need to earn money determined their choice in this matter.

“It is important that information regarding the workplace reaches all employees, and this does not always happen. Then I think, if I had the possibility and had a better economic situation, I would quit the job tomorrow.”

However, the employees expressed that they did not know how they would react until they were at the age of retirement, and they wanted their coworkers to support them to stay at work. The participants suggested that the management should offer duties adapted to older persons in order to keep them at work. It is important to handle and believe in each employee based on their personality and conditions and to let everyone be themselves. The employees were of the opinion that believing in each and everyone would promote health.

“The manager must believe in each and everyone and let them be themselves, that would promote everyone's health.”

Another important topic is to let the younger be involved in the older ones' work tasks. The participants reported that they had to let go and let the young employees show how they can manage the work tasks. The younger ones should not be seen as a threat for managing the older employees' work tasks better.

## 4. Discussion

This study contributes to novel perspectives of future challenges for companies and employees in older age. Employees are expected to work into older age and older employees might handle work tasks and stress in different ways, and this might lead to inequalities in workplace health.

Our results showed that the employees were of the opinion that changes in the workplace have a distinct impact on their health. This result is in line with Torp and Vinje [18], who concluded that it is important to consider the potential consequences when changing the workplace tasks or organisation. Furthermore, health could be affected when older employees have to work harder when other employees must quit working [18]. However, the results showed that a way to feel good at work, even if there were fewer employees in place, was to have access to temporary staff. Otherwise, there would be no time for reflection and stress could evolve, and this could lead to a risk for diseases [19]. To handle the stress, the employees had different strategies. One strategy was to train at the workplace's gym. However, this was not a solution for everyone, only for the persons who already were used to training. Not to join training could create a feeling of guilt and shame and thus contribute to more stress and illness [20]. In many societies today, there is an extreme pursuit of health with unhealthy training and eating habits, which make people feel bad instead of feeling good [21]. However, the employees had a solution and said that if they had access to coaches who could help them to handle changes in a way that was suitable for each employee, this could prevent illness and promote health for both older and younger employees.

Although the employees were aware that new technology was of importance in their work, they were also of the opinion that it was hard to learn how to use new technology. When introducing new technical duties, older employees must have time to learn at their own pace [22]. As another way to find balance at work, some of the employees had decided to work reduced hours when they got older, but this could also be stressful and would have to be handled [19]. To have a work balance, both physical and mental, there is a need for support from the OHS, and there is a need for more health promotion interventions instead of putting the focus only on preventing illness at workplaces [4].

The employees were of the opinion that everyone has the responsibility for their own health. However, they wanted the managers to help them to understand that they are doing a good job, thus suggesting that health is more than just physical activity. To feel good at work is more than just physical well-being [7], and this was confirmed by the coworkers in our study. They said that to train and to ensure physical well-being is the employee's own responsibility, but psychological well-being is also an issue for the managers at the company. To find the right balance is very important [23], and managers must ensure flexibility in working arrangements [24, 25].

Many of the employees were used to going to work even if they were sick. The employees also expressed that to work while sick might be a generational question, and they thought this was different for their younger coworkers. To be home from work while sick is a responsibility for both the employees themselves and the managers, and presenteeism—working while sick—must be discussed and guidelines regarding this is of importance [26, 27]. To take responsibility for the work to be done was of greater importance than to take responsibility for one's own health. Furthermore, the employees thought that working while sick was about relations to others, and to be home on sick leave might cause stress for their coworkers [28].

A salutogenic perspective has to be used when striving for good workplace health [4, 5]. Thus, in order to promote health, it is important that the individual will be seen and confirmed as a part of the team by both their coworkers and the managers. To be included, to feel needed, and to feel comfortable at work promote SOC, and this in turn promotes good health. Furthermore, learning to control the demands at work also strengthens one's SOC [29].

If they did not enjoy being at work, the employees said that they would not want to work for a long time at their workplace and would instead retire from work. The managers have a big responsibility to ensure that employees enjoy working and to work for a long time, but the employee's own motivation plays an important role [30]. It is an individual matter as to what an employee needs in order to be willing to work into old age, but to feel needed is a common factor [13, 14]. Therefore, managers have to handle each employee individually. However, this is also an economic question for the employees. If they have the economic possibility to quit working earlier and the work tasks and the work climate are too hard, they quit to work.

Other issues of importance for feeling needed at work were reported to be the need for physical contact. To hug someone or to be hugged is important for feeling good during the workday, and it is necessary for knowing that we see each other and are needed in the daily work [31]. This could be a health promotion “activity” to discuss, but it would need to be confirmed by all of the employees in the workplace.

In order to understand each other better at work, the employees in this study had participated in a course about different personalities and how to act in different situations. They said that this was experienced as promoting their own health [8].

To have support from the management is important for feeling good at work [31]. When getting older, one requires both support and tolerance from the management. The participants in this study also said that support from the OHS was needed. This has been shown in a previous study where managers and OHSC personnel reported that the OHS should work more with health promotion and not only with illness prevention [4].

The employees expressed how they did not know how they would react until they were at the age of retirement, and they wanted their coworkers to support them to stay at work instead of retiring. However, there must be flexibility regarding work tasks and a good work environment for older workers [24]. Shacklock and Brunetto [24] also showed other factors that are important for working longer, including the importance of the individual places on working and the individual's interests outside of work. The employees said that the management should offer duties adapted to older persons in order to keep them at work. It is important to handle and believe in each employee based on their personality and conditions and to be tolerant of everyone [32], and the employees in this study were of the opinion that believing in each and everyone would promote better health.

Another important topic is to let the young employees be involved. The younger employees should not be seen as a threat to the older employees. Older workers sharing their experiences and collaborating has been shown to be a successful way to promote health in the workplace [33].

When planning for working in later life, this study concludes with some thoughts and experiences but it would also be important to take gender into consideration. Loretto and Vickerstaff [25] showed that there are sometimes differences in flexible working conditions regarding gender and that this might affect the retirement process. Today, there is a need for competent employees with experiences of the company, and the management and one's coworkers should enable the possibilities for working into old age.

### 4.1. Strengths and Limitations

The purpose of this study was to explore employees' experiences of how to promote workplace health at the present time and for the end of working life. Therefore, we chose an exploratory design and a qualitative method and selected participants aged 50 years and older who were working in the company and were planning to continue working for the company into old age. To only include one workplace could be a limitation, but because it was a big company in an industrial setting with different work tasks, we obtained a wide variety of thoughts and experiences. However, it is of importance to take other settings into consideration because other settings of working life today is civil servants in healthcare, schools, and service sector and these employed face the same need for a healthy work into old age. In order to have a broad discussion, and because this issue is not very well researched, we used focus group interviews. Individual interviews would have been another choice, but with the findings from this study there is a better basis for directing individual questions for further research on this topic. We used a thematic analysis, which is a suitable method for analysing focus group discussions.

The findings in this study were evaluated in terms of trustworthiness, which could be demonstrated through credibility, dependability, and transferability [34]. Our study's credibility was strengthened by the process of choosing participants and the data collection method. Furthermore, the presentation of the analysis process and the quotations from the participants strengthen the credibility. To enhance the dependability, there were discussions between all of the researchers during the analysis (two nurses, one physiotherapist, and one economist). A careful description of the study population, data collection, and data analysis was provided, and this makes it possible to estimate the transferability of the study results.

## 5. Conclusions

The experiences regarding health promotion at work at the present time and for the end of working life were seen from employees' perspectives in an industrial setting. The findings showed that the employees have to handle changes in a changeable workplace and the employees meant that they need temporary staff when the work conditions changed. Further, the employees highlighted that both themselves and the managers at the workplace have to take responsibility for health concerns. There was also a need for confirmation for feeling needed and the employees meant that this was of importance for their health. Finally, support and tolerance have to be adapted for each employee in order to work longer. Promoting health should be an urgent mission for employees themselves as well as managers and the OHS in order to support employees in working into old age. The health promotion must be suitable for each employee and should be designed in such a way as to avoid inequality in workplace health between older and younger employees. Further research is needed in this area and from other settings such as civil servants in healthcare, schools, and service sector.

## Figures and Tables

**Table 1:** Process of the thematic analysis.

Phase 1: familiarisation	Data field notes and transcriptions were reflected in team meeting (ML, CH, AS, and KZ).

Phase 2: coding	ML, CH, AS, and KZ inductively coded the data. Both semantic and latent data were coded. Initially, 16 codes were identified; importance of good health, healthy living, exercise, healthy food, low stress, working condition, be needed, worries for computer-based working, ageing, changes in organisation, new project, uncertainty for the future, opportunities with senior life, checking own health, family structure, and social inclusion. The codes were triangulated in two small team meetings (ML, CH, AS, and KZ).

Phase 3: searching for themes	The process of searching for themes started in team meetings (ML, CH, AS, and KZ). An initial thematic map, detailed codes, and hierarchies of concepts were developed.

Phase 4: reviewing themes	ML, CH, AS, and KZ worked together in order to review and refine the five initial themes. All collated extracts were sorted and reviewed under each theme. During the process, two themes overlapped too much and were therefore reworked into four new themes.

Phase 5: defining and naming	The new themes were triangulated in team meetings (ML, CH, and KZ). Documentation of the team meetings gives consensus to the themes. Defining and naming four themes.

Phase 6: producing the report	ML, CH, AS, and KZ were checking the final version of the paper. All authors were involved in peer debriefing, interpretation data, coding, and analysis.

## Data Availability

Data field notes and transcriptions from the focus group interviews are available at Halmstad University.
